# Antimicrobial Resistance Patterns of *Escherichia coli* Isolated from Sheep and Beef Farms in England and Wales: A Comparison of Disk Diffusion Interpretation Methods

**DOI:** 10.3390/antibiotics10040453

**Published:** 2021-04-16

**Authors:** Charlotte Doidge, Helen West, Jasmeet Kaler

**Affiliations:** 1 School of Veterinary Medicine and Science, University of Nottingham, Sutton Bonington LE12 5RD, UK; charlotte.doidge@nottingham.ac.uk; 2 School of Biosciences, University of Nottingham, Sutton Bonington LE12 5RD, UK; helen.west@nottingham.ac.uk

**Keywords:** antibiotic resistance, sheep, beef cattle, *Escherichia coli*, normalised resistance interpretation, antimicrobial susceptibility testing, tetracyclines, farms

## Abstract

Little data exist on the levels of antimicrobial resistance from bacteria isolated from British sheep and beef cattle. The aim of this study was to investigate antimicrobial resistance patterns on sheep and beef farms in England and Wales using multiple interpretation methods. Fecal samples (*n* = 350) from sheep and beef cattle were collected from 35 farms. Disk diffusion antimicrobial susceptibility testing against ten antimicrobials was carried out for 1115 (699 sheep, 416 beef) β-glucuronidase-positive *Escherichia coli* isolates. Susceptibility was interpreted using clinical breakpoints, which determine clinically resistant bacteria, and epidemiological and livestock-specific cut-off values, which determine microbiological-resistant bacteria (non-wild type). Using livestock-specific cut-off values, a high frequency of wild type for all ten antimicrobials was observed in isolates from sheep (90%) and beef cattle (85%). Cluster analysis was performed to identify patterns in antimicrobial resistance. Interpretation of susceptibility using livestock-specific cut-off values showed a cluster of isolates that were non-wild type to cefotaxime and amoxicillin/clavulanic acid, whereas clinical breakpoints did not. A multilevel logistic regression model determined that tetracycline use on the farm and soil copper concentration were significantly associated with tetracycline non-wild type isolates. The results suggest that using human clinical breakpoints could lead to both the under-reporting and over-reporting of antimicrobial resistance in sheep and beef cattle.

## 1. Introduction

Antimicrobial resistance is a worldwide public health concern. The administration of antimicrobials leads to the selection of antimicrobial-resistant bacteria, and food-producing animals are one of several potential sources of antimicrobial resistance [1]. Although antimicrobial use is thought to be low in sheep and beef cattle [2,3], the large numbers of sheep and cattle in the UK may potentially contribute to the dissemination of antimicrobial-resistant bacteria [4,5]. National surveillance of antimicrobial resistance from bacteria isolated from sheep and beef cattle only uses samples that are submitted for clinical diagnostics [6]. The use of clinical isolates suggests that antimicrobial resistance to commonly used antimicrobials, such as tetracycline and ampicillin, is relatively high in sheep and cattle [7]. However, clinical samples are potentially biased as they usually come from sick animals which may have been treated with antimicrobials. At present in the UK, active national surveillance of healthy sheep or cattle does not exist.

There are few studies investigating antimicrobial susceptibility of organisms isolated from healthy sheep and beef cattle in the UK. These studies suggest that antimicrobial resistance on sheep and beef farms is relatively uncommon [8,9,10], although extended spectrum beta-lactamase (ESBL)-positive beef farms may be increasing [11,12]. Examining the presence of ESBL *E. coli* has been the focus of more recent studies on beef farms [11,12]. Therefore, other resistance types may have been missed in these studies. Other studies have investigated a larger range of antimicrobial resistances, but only investigated a few farms [8,10]. Hence, variance between farms with respect to antimicrobial resistance patterns was not investigated. More information regarding antimicrobial resistance on sheep and beef farms in the UK is required. Indeed, a systematic review of antimicrobial resistance on British sheep and cattle farms called for additional efforts in collecting farm-level antimicrobial resistance data [7].

Previously identified factors associated with antimicrobial resistance in pigs and veal calves in countries other than the UK include the use of antimicrobials, either as therapeutics to treat sick animals or as growth promoters [13,14,15]. Antimicrobial growth promoters are not used in the UK. The number of animals on the farm, region of the farm and type of animals sampled have also been reported as factors associated with antimicrobial resistance in bacteria isolated from animals [16,17]. It has been shown that bacterial isolates of animal origin may present with resistance even when the animals have not been exposed to antimicrobials [18,19]. Markland et al. [20] illustrated that when cefotaxime-resistant bacteria were present in samples from beef cattle, resistant bacteria were more abundant in soil samples. This indicated that the environment, such as soils and forage, may be a natural source of antimicrobial-resistant bacteria for food-producing animals [20]. However, the factors that affect the prevalence of antimicrobial-resistant bacteria in soils are unclear [21]. One potential explanation is that heavy metals such as copper and zinc may co-select for antimicrobial resistance in soil. The effect of metal concentrations in soil on the prevalence of antimicrobial resistance in farm animals requires further investigation.

Disk diffusion testing is a commonly used phenotypic method for determining antimicrobial susceptibility. Scientists typically interpret the results of such tests using clinical breakpoints and will mainly adhere to guidelines set by the European Committee on Antimicrobial Susceptibility Testing (EUCAST) or Clinical and Laboratory Standards Institute (CLSI) [22,23]. However, these clinical breakpoints are only relevant for human medicine [24]. CLSI has set very few veterinary clinical breakpoints, and at present, European veterinary breakpoints do not exist. Research suggests that using human clinical breakpoints to interpret veterinary data may lead to calculating a higher antimicrobial resistance prevalence than actually occurs [10].

An alternative method to interpret antimicrobial susceptibility data is to use epidemiological cut-off (ECOFF) values to determine fully susceptible isolates (wild type, WT) from non-fully susceptible isolates (non-wild type, NWT). EUCAST defines a WT organism as one with the absence of acquired and mutational resistance mechanisms to the drug in question [25]. Thus, the ECOFFs determine microbiological resistance, whereas clinical breakpoints determine clinical resistance. The ECOFF values are established by EUCAST through analysis of the distribution of their inhibitory zone diameters [26]. However, the distributions of inhibitory zone diameters for isolates of animal origin may differ from the distributions of inhibitory zone diameters for EUCAST isolates [10,27]. Therefore, ECOFF values may not reflect WT organisms isolated from livestock.

Instead, the normalised resistance interpretation (NRI) method can be used to calculate tailor-made cut-off values. The method was originally developed to calibrate the disk diffusion test to compare results between laboratories [28]. It has also been used to investigate the susceptibility of organisms of animal origin when EUCAST or CLSI breakpoints do not exist [29,30]. Furthermore, the NRI method has been used when clinical breakpoints or ECOFF values do not appear appropriate [10,27]. An inappropriate cut-off value occurs when the cut-off splits the normal distribution of inhibition zone diameters. Therefore, it may be useful to interpret the inhibitory zone diameters of isolates of animal origin using clinical breakpoints, ECOFF values and the NRI method so that comparisons can be made and the appropriate cut-off value can be chosen. A previous study based on isolates from four sheep farms compared these three interpretation methods and suggested that sheep-specific cut-off values were most fitting [10]. There needs to be additional studies with a larger number of participating farms to confirm these results, and similar studies have not been carried out for other livestock species. Additionally, the implications in terms of interpretation of antimicrobial resistance patterns requires further investigation. Therefore, the aim of this study was to investigate and compare antimicrobial resistance patterns on thirty-five sheep and beef farms in England and Wales using multiple interpretation methods, based on bacteria isolated from feces. Further objectives were to identify clusters of antimicrobial resistance and to identify factors that were associated with antimicrobial resistance on sheep and beef farms.

## 2. Results

The total number of isolates tested for each farm is presented in Table 1. A total of 1115 β-glucuronidase-positive *E. coli* isolates underwent antimicrobial susceptibility testing. Of these, 699 isolates were from 203 sheep fecal samples collected from 27 different farms, and 416 isolates were from 134 beef cattle fecal samples from 19 different farms.

### 2.1. Comparison of Methods to Interpret Resistance

The cut-off values determined by the NRI method (CO_WT_) for sheep and beef fecal derived isolates were larger for tetracycline compared with the clinical breakpoints (Table 2). The CO_WT_ values for sheep and beef were larger for ciprofloxacin, sulfamethoxazole-trimethoprim, cefotaxime and imipenem compared with the clinical breakpoints and ECOFF values. However, CO_WT_ values for sheep and beef were smaller for amoxicillin/clavulanic acid and ampicillin compared with the clinical breakpoints and ECOFF values. All CO_WT_ values had a standard deviation < 4.00 mm as recommended by Smith et al. [31]. CO_WT_ values with standard deviation between 3.36–4.00 mm were referred to as tentative CO_WT_ estimates. For beef cattle, four antimicrobials had tentative CO_WT_ estimates, and for sheep, two antimicrobials had tentative CO_WT_ estimates (Table 2).

Based on CO_WT_ values, 87.9% (980/1115) of all *E. coli* isolates were defined as WT organisms for all ten antimicrobials. Of the beef fecal isolates, 85.1% (354/416) were defined as WT for all ten antimicrobials. Of the sheep fecal isolates, 89.6% (626/699) were WT for all ten antimicrobials. The *E. coli* isolates had the lowest susceptibility to tetracycline, with 92.1% of sheep isolates being WT (Table 3) and 87.7% of beef isolates being WT (Table 4).

### 2.2. Farm-Level Susceptibility

All *E. coli* isolated from six farms were WT for the ten antimicrobials based on CO_WT_ values (6/35, 17%). Only 26% (9/35) of farms had all isolates WT to tetracycline, whereas all farms (35/35) had all isolates WT to imipenem (Table 5).

### 2.3. Cluster Analysis

**Figure 1:**
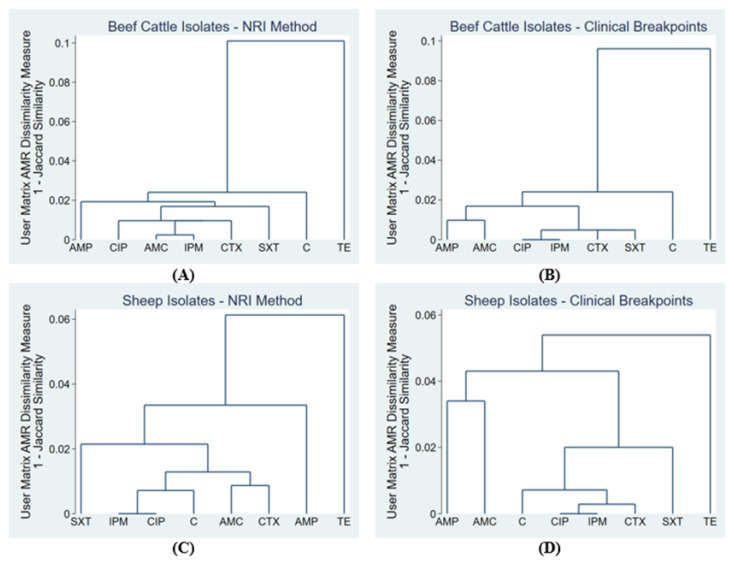
Single-linkage clustering dendrograms for non-susceptibility to eight antimicrobials (**A**) based on the NRI CO_WT_ values in *E. coli* isolates from beef cattle fecal samples (*n* = 416); (**B**) based on clinical breakpoints, in *E. coli* isolates from beef cattle fecal samples (*n* = 416); (**C**) based on the NRI CO_WT_ values, in *E. coli* isolates from sheep fecal samples (*n* = 699); and (**D**) based on clinical breakpoints, in *E. coli* isolates from sheep fecal samples (*n* = 699). AMP = ampicillin, AMC = amoxicillin/clavulanic acid, C = chloramphenicol, CIP = ciprofloxacin, CTX = cefotaxime, IPM = imipenem, SXT = sulfamethoxazole-trimethoprim, TE = tetracycline.

The dendrograms from the single-linkage cluster analysis of susceptibility to eight antimicrobials in *E. coli* isolates from beef cattle fecal samples and sheep fecal samples are presented in Figure 1.

For both the sheep and beef isolates, the cluster analyses using clinical breakpoints identified a cluster of isolates that were non-susceptible to ampicillin and amoxicillin/clavulanic acid. This cluster was not identified when using the NRI CO_WT_ values. In all four cluster analyses, tetracycline was the least related to other antimicrobial susceptibilities. A cluster of sheep isolates non-wild type for cefotaxime and amoxicillin/clavulanic acid was identified using NRI CO_WT_ values, but not using clinical breakpoints.

### 2.4. Multilevel Logistic Regression Model

#### 2.4.1. Base Model

The base multilevel logistic regression model indicated that 16% of the variance of an isolate being defined as non-wild type for tetracycline was due to between-farm differences and 76% of the variance was due to between-sample differences.

#### 2.4.2. Univariable Multilevel Logistic Regression Models

A univariable multilevel logistic regression analysis was carried out to determine potential factors associated with the presence of tetracycline non-wild type isolates. Table 6 presents the associations of potential risk factors.

#### 2.4.3. Multivariable Multilevel Logistic Regression Model

The odds of isolates being defined as non-wild type for tetracycline were 28 times higher (CrI = 2.50–520.09) when farms used tetracycline antimicrobials in their animals (Table 7). With every standardised unit increase for soil copper concentration, the odds of isolates being defined as non-wild type for tetracycline was 1.78 times higher (CrI = 1.02–3.21).

The multivariable multilevel logistic regression model indicated that 9% of the residual variance of an isolate being defined as non-wild type for tetracycline was due to between-farm differences and 76% of the residual variance was due to between-sample differences.

## 3. Discussion

In this study, antimicrobial resistance patterns in *E. coli* isolated from sheep and beef farms were assessed using different interpretation methods. The results show that antimicrobial resistance in feces on sheep and beef farms in England and Wales is generally low regardless of interpretation method, compared with samples from other livestock species in the UK [6]. However, interpretation method can have important implications on the understanding of resistance patterns. Different clusters of resistance patterns were determined when using clinical breakpoints compared with bespoke cut-off values using the NRI method.

The comparison of susceptibility interpretation methods showed that differences in the proportion of susceptible isolates may occur depending on the method used. There was little correlation between interpretations of susceptibility using clinical breakpoints, ECOFFs and CO_WT_ values for amoxicillin/clavulanic acid in particular. For the beef isolates, there was also little correlation between the interpretations for ciprofloxacin and sulfamethoxazole-trimethoprim. This is possibly because clinical breakpoints detect human clinical resistance, whereas ECOFFs and CO_WT_ values detect microbiological resistance. The results suggest that clinical breakpoints and ECOFFs are not appropriate for interpreting antimicrobial resistance in bacteria isolated from sheep or cattle feces as they do not fit the wild type distribution of isolates. Silva et al. [10] also showed that clinical breakpoints and ECOFFs may be inappropriate for the classification of ovine isolates as resistant and that these interpretation methods may over-report antimicrobial resistance in sheep populations [10]. Our results also indicate that clinical breakpoints and/or ECOFFs may slightly under-report, as well as over-report, antimicrobial resistance in sheep and beef isolates, particularly for sulfamethoxazole-trimethoprim, tetracycline and cefotaxime.

Silva et al. [10] further suggest that the sheep industry could establish sheep-specific cut-offs to avoid over-interpretation of resistance in ovine isolates. It is important to highlight that some of the sheep-specific cut-offs determined in the study by Silva et al. [10] were vastly different to those determined in our study. For example, Silva et al. [10] calculated the sheep CO_WT_ for tetracycline to be 14 mm, whereas we calculated a sheep CO_WT_ of 25 mm. The difference could be because of differences in the study population. The present study investigated sheep samples from 27 farms in England and Wales, whereas Silva et al. [10] used samples from just three farms in Scotland and one farm in Norway. Additionally, some isolates collected by Silva et al. [10] were from diseased animals and not from fecal samples. Nevertheless, this demonstrates the need for large-scale data collection from a variety of different farms before industry-wide cut-off values can be developed. These results also demonstrate the need to use the NRI method to calibrate the disk diffusion test to compare results between laboratories.

The differences in interpretation methods led to disparities in the groupings of antimicrobial resistances through cluster analysis. There was a cluster of isolates that were non-susceptible to ampicillin and amoxicillin/clavulanic acid for both the sheep and beef samples when the clinical breakpoints were used, but were not clustered as non-wild type when CO_WT_ values were used. A cluster of sheep isolates that were non-wild type for cefotaxime and amoxicillin/clavulanic acid was only determined with the CO_WT_ values. This may have implications for elucidating resistance mechanisms. Non-susceptibility to amoxicillin/clavulanic acid and ampicillin or cefotaxime suggests mechanisms of beta-lactam resistance, which requires further investigation through genotypic analysis [32]. These non-susceptibility and non-wild type patterns may have been missed if only one interpretation method was used.

A few cases of cefotaxime non-susceptibility in sheep and beef fecal isolates were reported in this study. Reduced susceptibility to cefotaxime has been reported in British beef cattle [12]. Although cefotaxime resistance in isolates from sheep has previously been reported in England and Wales, this was from clinical diagnostic samples [11], and to the authors’ knowledge, it has not been reported for apparently healthy sheep in England and Wales before. This may be because third-generation cephalosporins are very rarely used on sheep farms. The use of highest-priority critically important antimicrobials should only be used as a last resort, when susceptibility testing has been conducted and no other antimicrobial would be effective. As resistance to other lower priority antimicrobials is uncommon in sheep and beef cattle, the use of third-generation cephalosporins is usually not required. It has previously been shown that cefotaxime-resistant bacteria may be present on beef farms without any antimicrobial use and that the environment may be a source of cefotaxime resistance [19,20]. The presence of cefotaxime non-susceptibility raises concerns around the existence of extended spectrum beta-lactamases (ESBLs) in healthy sheep and beef cattle [32], especially as a group of sheep isolates resistant to both cefotaxime and amoxicillin/clavulanic acid was identified from the cluster analysis. Further screening of the cefotaxime non-susceptible isolates is required to determine the presence of ESBL resistance mechanisms [33].

The results suggest that antimicrobial resistance to commonly used antimicrobials (such as tetracycline and penicillin) in apparently healthy sheep and beef cattle in England and Wales is much lower than that reported from national clinical surveillance [6,34]. This is probably because clinical samples are more likely to come from sick animals that have already been treated with antimicrobials before submission. Additionally, beef and dairy cattle samples are often not separated when reporting antimicrobial resistance [6]. In apparently healthy dairy cattle, the proportion of resistant isolates is much higher than the beef cattle reported here [35]. Although antimicrobial resistance appears to be low on sheep and beef farms in England and Wales, these figures were higher than what was reported historically. In 1999, 3% of isolates from sheep, and 6% of isolates from cattle were resistant to one or more antimicrobials [9]. In contrast, in our study, 10% of isolates from sheep, and 15% of isolates from cattle were non-wild type for one or more antimicrobials. Similarly, less than 4% of *E. coli* isolates from Scottish beef farms between 2001 and 2004 were resistant to tetracycline [8], compared with 12% of beef isolates in our study. This difference in susceptibility may be due to differences in study design, for example, differences in the antimicrobials studied or sampling technique. Additionally, the use of different interpretation methods may play a role in the varying antimicrobial susceptibilities as an organism that is classed as non-wild type does not necessarily display clinical resistance. Alternatively, differences in susceptibilities between the studies may be due to changes in farm practices over the last twenty years. This highlights the need for regular and consistent surveillance of antimicrobial resistance on sheep and beef farms in the UK so that longitudinal comparisons can be made.

The majority of the variance in antimicrobial susceptibility between isolates was due to between-sample differences, whereas only a small proportion of variance was due to between-farm differences. Around half of the between-farm variance could be explained by the use of tetracycline on the farm and the concentration of copper in the soils; however, there was still a large proportion of between-sample variation that was unexplained. This suggests that differences in antimicrobial resistance patterns are due to the variability in management of individual animals rather than any whole flock or herd management practices. The results probably reflect that antimicrobials are not usually used as routine prophylactic (preventative) treatments on sheep and beef farms in the UK, and in most cases farmers only use antimicrobials for the treatment of sick individual animals [3,36]. This is encouraging as there has been a large push in the UK agriculture industry to voluntarily reduce antimicrobial use over the last five years, particularly targeted at whole flock and herd treatments [36,37]. The large sample-level variance may also be due to the individual characteristics of the animals. For example, cattle over the age of 25 months have been shown to carry significantly less antimicrobial-resistant *E. coli* compared with younger cattle [17]. It was not possible to gather much sample-level information in this study as fecal samples were collected from the ground rather than directly from the animals. To understand the sources of variance at the sample level, further investigation using samples obtained directly from individual animals is recommended. The high variability between samples suggests that future studies that aim to understand the drivers of antimicrobial resistance on farms should consider taking individual animal samples rather than pooled samples.

The use of phenicols and tetracyclines was significantly associated with tetracycline non-wild type isolates in the univariable analysis. The association between tetracycline use and tetracycline non-wild type isolates is not surprising and has previously been reported for other livestock species [13,15]. The use of a particular antimicrobial will result in the direct selection of the corresponding resistance [38]. Phenicol use might indirectly select for tetracycline resistance via cross-resistance mechanisms. Alternatively, phenicol use may be associated with tetracycline non-wild type isolates if farmers change their antimicrobial drug of choice from tetracycline to phenicol when they find tetracyclines are no longer as effective for them. Tetracyclines are used as first-line treatments for livestock in the UK, whereas phenicols are in a higher category of antimicrobial which should only be used when first-line treatments are unavailable, for example, in cases of clinical tetracycline resistance [39]. Other factors that were identified as significant influences on antimicrobial resistance in previous studies, such as weather [35] and farm size [17], were not significant in our study. One possible reason for this is that for the dependent variable, there was only a small proportion of non-wild type isolates for tetracycline.

The concentration of copper in the soils in the farm area and the use of tetracyclines were significantly associated with tetracycline non-wild type isolates in the multivariable analysis. Previous research indicates that the geochemical conditions of soils, particularly copper concentrations, are correlated with the abundance of antimicrobial resistance genes [40,41]. Furthermore, copper has been shown to co-select for tetracycline resistance in experimental conditions [42], and mathematical models suggest that this co-selection may occur at copper concentrations as low as 5.5 mg/mL [43]. Our results suggest that tetracycline non-susceptibility may be more prevalent on some farms due to the environmental exposure to copper in soils. There needs to be further investigation into the presence of copper resistance genes in the tetracycline non-wild type isolates obtained in this study.

### Limitations

The study sample was small; however, the number of farms was comparable to that of similar studies investigating antimicrobial resistance at the farm level and the farms represented a range of farm types and sizes [44,45]. Additionally, the farms were mainly located in Wales and the West of England, where sheep and cattle are more densely populated [46,47]. This was a cross-sectional study and so changes in antimicrobial resistance over time could not be measured. Additionally, information was mainly only collected at the farm level, but analysis indicated that most of the variation in resistance was at the sample level. Further investigation using a longitudinal study design and collecting sample-level information from individual animals is required to understand additional variation in resistance levels on farms. The β-glucuronidase-positive *E. coli* isolates were the focus here, although other species not studied are likely to also be environmentally important.

## 4. Materials and Methods

### 4.1. Participant Recruitment

Farmers identified for participation in the study were those that had previously completed a sheep flock health survey, beef herd health survey or both. The survey was distributed by a British retailer; therefore, all farms participating in this study supply to the retailer. Those that indicated that they would be interested in sharing their antimicrobial use data in the survey were contacted by their preferred form of contact, either telephone or email. Farmers were contacted in order of preference until thirty-five farms were recruited. Preference was farms that had sheep and/or cattle numbers that were representative of the producer average based on previous survey data [3].

The study was approved by the University of Nottingham School of Veterinary Medicine and Science Ethics Committee (No. 1850 160916).

### 4.2. Sample Collection

Each farm was visited between February and October 2019. Ten fecal samples from either sheep or beef cattle were taken at each farm. Random samples of fresh feces were taken from the field or pen floor and each placed into a sterile bag. Previous research shows that taking samples from the floor provides similar antimicrobial resistance profiles to taking samples directly from the rectum of the animals [48,49]. Therefore, samples from the floor were taken to reduce stress on the animals. Location of sample collection was not the same for each farm due to the different production systems. Therefore, sample collection location was recorded (e.g., indoor pen or outdoor pasture). Farmers were asked if they thought any of the animals in the field/pen had been given antimicrobials in the past two weeks. If so, the fecal samples were excluded from further analysis. The samples were kept cool and were processed within 24 h at laboratories at the University of Nottingham.

### 4.3. Isolation of Escherichia coli

For each sample, 2 g of feces was weighed and suspended in 18 mL of Maximum Recovery Diluent (MRD) (Oxoid). Samples were serially diluted, and 200 µL aliquots were plated onto Tryptone Bile X-glucuronide (TBX) agar (Oxoid) for the detection of β-glucuronidase-positive *E. coli*. *E. coli* form blue colonies on these plates while other Enterobacteriaceae form white colonies. TBX plates were incubated at 35 °C for 24 h. For each sample, the number of colonies with a typical *E. coli* phenotype were counted. The plate with between 30 and 300 colonies was chosen, and six blue colonies from each plate were picked for streak plating. Single colonies were streaked onto Luria-Bertani (LB) agar (Lennox) and incubated at 35 °C for 24 h. From each plate, a single colony was put into a Microbank (Pro-Lab Diagnotics UK) and placed in a −80 °C freezer.

### 4.4. Antimicrobial Susceptibility Testing

At least three isolates from each sample were chosen for antimicrobial susceptibility testing. Antimicrobial susceptibility testing was undertaken following the EUCAST guidelines [22]. Antimicrobial resistance testing was carried out for ten antimicrobials using the disk diffusion method. Colonies were suspended in MRD until it reached 0.5 McFarland standard. The dilution was then streaked across a Mueller-Hinton Agar plate (Oxoid), and the 10 antimicrobial disks were placed onto the surface of the agar. The plates were incubated for 24 h at 35 °C. The zone diameters were then recorded using the EUCAST guidelines where possible [24]. For antimicrobials that do not have EUCAST breakpoints available, CLSI guidelines were followed [23]. All antimicrobials used are shown in Table 8 and were supplied by Oxoid (Basingstoke, UK).

### 4.5. Terminology

Epidemiological cut-off values determined by EUCAST are referred to by the acronym ECOFF. For differentiation from the ECOFF values, the sheep-specific and beef-specific cut-off values determined by the NRI method in this study are referred to by the acronym sheep CO_WT_ and beef CO_WT_, respectively.

CO_WT_ and ECOFF values determine WT and NWT organisms, where WT organisms are characterised as devoid of phenotypically detectable acquired resistance mechanisms. The term “resistant” is reserved for clinically resistant organisms. Clinical breakpoints determine susceptible (S) and resistant (R) organisms, where S organisms are characterised by a level of antimicrobial activity associated with a high likelihood of therapeutic success [25].

### 4.6. Data Analysis

Data cleaning, descriptive analysis, cluster analyses and univariable multilevel logistic regression modelling were carried out in Stata software (Stata SE/16.1, Stata Corp., College Station, TX, USA). The isolates obtained from three samples from Farm 2 were excluded from analysis as the sampled sheep were recently administered antimicrobials. The data used for this study are available in the Supplementary Material.

#### 4.6.1. Determining Cut-Off Values

Clinical breakpoints, ECOFFs and CO_WT_ values were used to determine the proportion of fully susceptible/wild type organisms. The normalised resistance interpretation (NRI) method was used to calculate sheep CO_WT_ and beef CO_WT_. NRI is based on the assumption that the wild type distribution is normal. The mean and the standard deviation are calculated from a plot of probit values of their cumulative frequencies of observations against their respective susceptibility measures [28]. The CO_WT_ was determined as 2.5 standard deviations from the mean. The automatic and manual Excel programs used to calculate the CO_WT_ were made available through courtesy by P. Smith, W. Finnegan and G. Kronvall [50].

Upon inspection of the disk plots produced using the NRI automatic cut-off calculator, the calculated cut-offs for ciprofloxacin and chloramphenicol for sheep and imipenem and chloramphenicol for beef were deemed inappropriate. This was because of an outlying peak in the high-zone part. For these four NRI calculations, the manual NRI calculator was used instead. Usually, the first drop in the rolling means determines the putative peak used for the NRI calculations, whereas if there was an outlying peak in the high-zone part, the second drop in the rolling means was used to determine the putative peak [28].

The NRI method was used with permission from the patent holder, Bioscand AB, TÄBY, Sweden (European patent No 1383913, US Patent No. 7,465,559).

#### 4.6.2. Descriptive Statistics

The proportion of susceptible/wild type *E. coli* isolates was calculated for each antimicrobial used for the susceptibility testing. The kappa-statistic was used to measure the level of agreement between the interpretations of clinical breakpoints, ECOFFs and CO_WT_ values. A score of 1 indicates perfect agreement, and a score of 0 indicates the amount of agreement that would be expected to be observed by chance [51]. The proportion of farms that had full susceptibility/wild type to each antimicrobial was also calculated.

#### 4.6.3. Cluster Analysis

To determine potential groupings in antimicrobial resistances, single-linkage hierarchical agglomerative clustering was implemented [52]. The Jaccard similarity measure was used to compare antimicrobial susceptibility for ten antimicrobials, saving one minus the Jaccard measure as a dissimilarity matrix. Cluster analysis was performed four times to produce dendrograms for antimicrobial susceptibility for (1) sheep isolates using the CO_WT_ values, (2) sheep isolates using clinical breakpoints, (3) beef isolates using CO_WT_ values and (4) beef isolates using clinical breakpoints. A low dissimilarity measure indicated that two antimicrobial susceptibilities were related. A dissimilarity measure of zero indicated that all isolates were susceptible to both antimicrobials.

#### 4.6.4. Multilevel Logistic Regression Base Model

A binary variable for tetracycline non-wild type (based on CO_WT_ values) was chosen as the dependent variable as this antimicrobial had the lowest proportion of antimicrobial susceptibility for both sheep and beef isolates. Multilevel logistic regression was not performed for the other antimicrobials because there were very few isolates that were non-susceptible to the other antimicrobial families. A base model with no predictor variables and three levels was run. If $y_{\mathrm{ijk}}$ = 1 if the ith isolate from sample j from farm k is non-wild type for tetracycline and $y_{\mathrm{ijk}}$ = 0 if it is wild type for tetracycline, then we model:

(1) $$y_{\mathrm{ijk}}\sim \mathrm{Bernouilli}\left(\pi_{\mathrm{ijk}}\right)$$

(2) $$\mathrm{logit}\left(\pi_{\mathrm{ijk}}\right){=\beta +v}_{k}{+u}_{\mathrm{jk}}$$

(3) $$v_{k}=N\left({0,\sigma }_{v}^{2}\right){,\;u}_{\mathrm{jk}}{\sim N(0,\sigma }_{u}^{2})$$

where β is the probability of the isolate being non-wild type, $u_{\mathrm{jk}}$ is the sample effects (with variance $\sigma_{u}^{2}$) and $v_{k}$ is the farm effects (with variance $\sigma_{v}^{2}$).

The intraclass correlation coefficients were determined to understand the underlying variation in susceptibility at the sample and farm level.

#### 4.6.5. Multivariable Multilevel Random-Intercept Logistic Regression

Additional data were collected to investigate risk factors that might be associated with tetracycline non-susceptibility, based on the findings of previous research [13,14,16,20]. Meteorological data were extracted from UK Meteorological Office data [53]. The average maximum and minimum temperature (°C), and rainfall (mm) from the closest weather station for the month of sample collection were recorded for each farm. Soil data were extracted from the UK Soil Observatory based on the postcode of each farm [54]. Antimicrobial use data were collected using the bin method [55]. In a visit prior to the sample collection, farms were instructed to place any empty antimicrobial packaging used in sheep/beef cattle into a bin. The contents of the bin were collected during the sampling visit. From this, a binary variable for the presence/absence of each antimicrobial class was produced.

A univariable three-level logistic regression analysis was carried out to determine associations with isolates being tetracycline non-wild type. Variables with *p* ≤ 0.2 were considered for the multivariable three-level random-intercept logistic regression model. The multivariable multilevel logistic regression model was fitted in MLwiN 3.02 [56]. Initially, model exploration was conducted using first-order marginal quasi-likelihood. Then, Markov-chain Monte Carlo (MCMC) simulations using Metropolis–Hastings sampling with diffuse priors, a burn-in length of 5000 and a run of 50,000 iterations were used to fit the multivariable model. The Raftery–Lewis diagnostic suggested that a Markov chain of at least 435,000 was needed to estimate the 2.5% quantile for the intercept coefficient. Therefore, MCMC simulations with a burn-in length of 10,000 and a run of 500,000 iterations were used to fit the final multivariable multilevel logistic regression model. A forward and backward selection stepwise model-building approach was used, where only variables with *p* ≤ 0.05 were selected to remain in the model. If $y_{\mathrm{ijk}}$ = 1 if the ith isolate from sample j from farm k is non-wild type to tetracycline and $y_{\mathrm{ijk}}$ = 0 if it is wild type to tetracycline, then we model:

(4) $$y_{\mathrm{ijk}}\sim \mathrm{Bernouilli}\left(\pi_{\mathrm{ijk}}\right)$$

(5) $$\mathrm{logit}\left(\pi_{\mathrm{ijk}}\right){=\beta }_{0}{+\beta }_{1}{\mathrm{TetracyclineUse}}_{\mathrm{ijk}}{+\beta }_{2}{\mathrm{CuConc}}_{\mathrm{ijk}}{+v}_{k}{+u}_{\mathrm{jk}}$$

(6) $$v_{k}=N\left({0,\sigma }_{v}^{2}\right),\;u_{\mathrm{jk}}{\sim N(0,\sigma }_{u}^{2})$$

where $\beta_{0}$ is the intercept; $\beta_{1}$ is the coefficient for the effect of a unit increase of the predictor ${\mathrm{TetracyclineUse}}_{\mathrm{ijk}}$ on the outcome; $\beta_{2}$ is the coefficient for the effect of a unit increase of the predictor ${\mathrm{CuConc}}_{\mathrm{ijk}}$ on the outcome; and $v_{k}$ and $u_{\mathrm{jk}}$ are the random effects at the farm and sample level, respectively.

The variance partitioning coefficients were calculated under the latent variable method, which assumes the binary outcome arises from an underlying continuous distribution and that the level 1 variance on the logit scale is π^2^/3 [57].

(7) $${\mathrm{VPC}}_{k}{=\sigma }_{v}^{2}/{(\sigma }_{v}^{2}{+\sigma }_{u}^{2}{+\pi }^{2}/3)$$

(8) $${\mathrm{VPC}}_{j}{=(\sigma }_{v}^{2}{+\sigma }_{u}^{2})/{(\sigma }_{v}^{2}{+\sigma }_{u}^{2}{+\pi }^{2}/3)$$

where VPC_k_ is the VPC at the farm level, VPC_j_ is the VPC at the sample level, $\sigma_{v}^{2}$ is the variance at the farm level, and $\sigma_{u}^{2}$ is the variance at the sample level.

Selection of the best fitting model was based on the value of Bayesian Deviance Information Criterion (DIC). The model with the lowest DIC value was considered the best fitting model.

## 5. Conclusions

Antimicrobial non-susceptibility of *E. coli* isolated from healthy sheep and beef cattle in England and Wales appears to be low compared with reports from clinical diagnostic isolates. However, antimicrobial non-susceptibility from healthy animals may have increased in the past twenty years. The use of tetracyclines on farms and environmental copper exposure in soils may contribute to tetracycline resistance. Uniform methods of antimicrobial susceptibility testing are required to make longitudinal comparisons and monitor long-term changes in resistance patterns. Using human clinical breakpoints could lead to the under-reporting and over-reporting of antimicrobial resistance in sheep and beef cattle. The use of livestock-specific cut-off values for interpreting antimicrobial susceptibility can provide more appropriate estimates of susceptibility for sheep and beef cattle.

## Figures and Tables

**Table 1:** Description of the farms where *E. coli* isolates were obtained including number of animals, region and number of isolates tested.

Farm No.	Region	*n* Beef Cattle (All Ages)	*n* Ewes	*n* Sheep Isolates	*n* Beef Isolates
1	West Midlands	220	0	0	30
2	West Midlands	205	370	10	15
3	West Midlands	281	900	13	16
4	Wales	125	750	15	16
5	South West England	2240	0	0	32
6	West Midlands	172	350	25	15
7	West Midlands	342	0	0	32
8	South West England	500	0	0	36
9	South West England	218	1058	33	0
10	Wales	236	840	30	0
11	Wales	93	550	26	17
12	Wales	0	250	15	0
13	Wales	109	584	30	0
14	South East England	198	800	10	13
15	Wales	39	538	28	0
16	Wales	41	500	39	0
17	Wales	179	1850	15	15
18	Wales	600	800	15	15
19	West Midlands	107	0	0	30
20	Wales	161	582	30	0
21	West Midlands	0	300	39	0
22	South West England	49	480	29	0
23	West Midlands	157	520	40	0
24	South West England	64	560	30	0
25	South West England	209	600	29	0
26	North East England	200	500	25	15
27	North West England	420	0	0	30
28	South West England	241	0	0	30
29	Wales	0	300	28	0
30	Wales	145	466	27	15
31	Wales	23	360	39	0
32	Wales	564	1600	15	15
33	West Midlands	285	0	0	29
34	West Midlands	0	600	31	0
35	Wales	40	425	33	0

*n* = number.

**Table 2:** Epidemiological cut-off values calculated using the NRI method compared with clinical breakpoints and ECOFF values.

Antimicrobials	Disk Content	Clinical Breakpoint (S ≥ mm)	ECOFF WT ≥ mm	Sheep CO_WT_ WT ≥ mm	SD	Beef CO_WT_ WT ≥ mm	SD
Neomycin	30 μg	-	-	13	1.46	14	1.87
Spectinomycin	100 μg	-	-	19	1.91	18	2.06
Tetracycline	30 μg	15	-	25	2.25	26	2.17
Amoxicillin/Clavulanic Acid	20–10 µg	19	16	15	3.15	15 *	3.66
Ciprofloxacin	5 µg	25	25	27 *	3.72	32	2.42
Ampicillin	10 µg	14	14	12	3.26	11 *	3.61
Sulfamethoxazole-Trimethoprim	23.75–1.25 µg	14	21	24	2.96	24	2.72
Chloramphenicol	30 µg	17	17	18 *	3.50	17 *	3.65
Cefotaxime	5 µg	20	21	26	2.26	26	3.04
Imipenem	10 µg	22	24	27	2.82	27 *	3.88

* SD > 3.34 mm and therefore CO_WT_ only a tentative estimate. S = susceptible, WT = wild type.

**Table 3:** Prevalence of antimicrobial susceptible (S) and wild type (WT) *E. coli* isolated from sheep using clinical breakpoints, ECOFFs and the NRI method.

Antimicrobial	*n* Isolates Sheep	Clinical Breakpoint (% S)	ECOFF (% WT)	Sheep CO_WT_ (% WT)	Kappa
Neomycin	699	-	-	99.6%	N/A
Spectinomycin	699	-	-	95.9%	N/A
Tetracycline	699	93.0%	-	92.1%	0.938
Amoxicillin/Clavulanic Acid	699	95.4%	97.4%	98.1%	0.689
Ciprofloxacin	699	100%	100%	100%	N/A
Ampicillin	699	94.7%	94.7%	95.1%	0.971
Sulfamethoxazole-Trimethoprim	699	98.0%	98.0%	97.9%	0.976
Chloramphenicol	699	99.3%	99.3%	99.3%	1.000
Cefotaxime	699	99.7%	99.1%	98.7%	0.585
Imipenem	699	100%	100%	100%	N/A

**Table 4:** Prevalence of antimicrobial susceptible (S) and wild type (WT) *E. coli* isolated from beef cattle using clinical breakpoints, ECOFFs and the NRI method.

Antimicrobial	*n* Isolates Beef	Clinical Breakpoint (% S)	ECOFF (% WT)	Beef CO_WT_ (% WT)	Kappa
Neomycin	416	-	-	100%	N/A
Spectinomycin	416	-	-	99.0%	N/A
Tetracycline	416	88.2%	-	87.7%	0.977
Amoxicillin/Clavulanic Acid	416	98.3%	99.5%	99.8%	0.395
Ciprofloxacin	416	99.8%	99.8%	99.0%	0.423
Ampicillin	416	97.8%	97.8%	97.8%	1.000
Sulfamethoxazole-Trimethoprim	416	99.5%	99.5%	98.1%	0.495
Chloramphenicol	416	97.6%	97.6%	97.6%	1.000
Cefotaxime	416	99.5%	99.3%	99.0%	0.776
Imipenem	416	100%	100%	100%	N/A

**Table 5:** Farm-level prevalence of antimicrobial susceptibility of all *E. coli* isolated from sheep and beef farms based on the NRI method (CO_WT_) and clinical breakpoints.

Antimicrobial	Farms Having All Isolates as Wild Type	
	CO_WT_	Clinical breakpoint
Neomycin	33/35 (94%)	-
Spectinomycin	21/35 (60%)	-
Tetracycline	9/35 (26%)	10/35 (29%)
Amoxicillin/Clavulanic Acid	30/35 (86%)	18/35 (51%)
Ciprofloxacin	32/35 (91%)	34/35 (97%)
Ampicillin	17/35 (49%)	16/35 (46%)
Sulfamethoxazole-Trimethoprim	22/35 (63%)	26/35 (74%)
Chloramphenicol	27/35 (77%)	27/35 (77%)
Cefotaxime	31/35 (86%)	32/35 (91%)
Imipenem	35/35 (100%)	35/35 (100%)

**Table 6:** Univariable multilevel logistic regression analysis for risk factors associated with *E. coli* defined as non-wild type for tetracycline.

Factor	Unit	*n*	Odds Ratio (95% CI)	*p*-Value
Flock size	*n* ewes	1115	0.91 (0.46, 1.81)	0.792
Herd size	*n* cattle > 12 months	1115	0.90 (0.44, 1.82)	0.761
Region: Wales	No	737		
	Yes	378	1.62 (0.37, 7.11)	0.523
Region: West Midlands (England)	No	790		
	Yes	325	1.08 (0.23, 5.13)	0.924
Region: Southern England	No	823		
	Yes	242	0.39 (0.07, 2.25)	0.292
Indoor samples	No	568		
	Yes	547	2.90 (0.77, 10.97)	0.116
Mixed species farm	No	362		
	Yes	753	1.93 (0.41, 9.09)	0.404
Animal species sample origin	Cattle	416		
	Sheep	699	0.38 (0.11, 1.28)	0.118
Maximum average temperature of sampling month	°C	1115	0.90 (0.45, 1.79)	0.760
Minimum average temperature of sampling month	°C	1115	0.79 (0.49, 1.95)	0.939
Average rainfall in sampling month	mm	1115	1.23 (0.62, 2.42)	0.556
Tetracycline use	No	159		
	Yes	956	22.21 (1.46, 337.52)	0.026
Penicillin use	No	157		
	Yes	958	0.62 (0.09, 4.44)	0.635
Aminoglycoside use	No	363		
	Yes	752	0.52 (0.12, 2.19)	0.376
Macrolide use	No	634		
	Yes	481	1.95 (0.48, 7.90)	0.384
Phenicol use	No	825		
	Yes	290	6.98 (1.82, 26.80)	0.005
Sulphonamide use	No	993		
	Yes	122	2.60 (0.32, 21.03)	0.371
Soil copper concentration	mg/kg	1115	1.72 (0.97–3.05)	0.062
Soil zinc concentration	mg/kg	1115	0.90 (0.45, 1.78)	0.755
Soil lead concentration	mg/kg	1115	1.60 (0.85, 3.00)	0.144
Soil cobalt concentration	mg/kg	1115	0.65 (0.34, 1.26)	0.206

**Table 7:** Multivariable multilevel logistic regression analysis for risk factors associated with *E. coli* isolates being defined as non-wild type for tetracycline.

Variable	Unit	*n*	Odds Ratio (95% CrI *)	*p*-Value
Tetracycline use	No	159		
	Yes	956	28.22 (2.50, 520.09)	0.014
Soil copper concentration	mg/kg	1115	1.78 (1.02, 3.21)	0.046
**Random Effects**			Variance Estimate (95% CrI *)	
Farm			1.24 (0.003, 4.47)	
Sample			9.36 (4.86, 16.38)	

* CrI = credible interval.

**Table 8:** Disk contents used to determine antimicrobial susceptibility of *E. coli* isolates and source of clinical breakpoints.

Antimicrobials	Disk Content	Source
Neomycin	30 μg	N/A
Spectinomycin	100 μg	N/A
Tetracycline	30 μg	CLSI
Amoxicillin/Clavulanic Acid	20–10 µg	EUCAST
Ciprofloxacin	5 µg	EUCAST
Ampicillin	10 µg	EUCAST
Sulfamethoxazole-Trimethoprim	23.75–1.25 µg	EUCAST
Chloramphenicol	30 µg	EUCAST
Cefotaxime	5 µg	EUCAST
Imipenem	10 µg	EUCAST

## Data Availability

The data presented in this study are available in the Supplementary Material.

## Supplementary Materials

The data used for this study are available in the Supplementary Material available online at https://www.mdpi.com/article/10.3390/antibiotics10040453/s1.
